# Potential Cross-Reactive Immunity to SARS-CoV-2 From Common Human Pathogens and Vaccines

**DOI:** 10.3389/fimmu.2020.586984

**Published:** 2020-10-16

**Authors:** Pedro A. Reche

**Affiliations:** Department of Immunology & O2, Faculty of Medicine, University Complutense of Madrid, Madrid, Spain

**Keywords:** coronavirus disease 19 (COVID-19), severe acute respiratory syndrome coronavirus 2 (SARS-CoV-2), epitope, cross-reactive immunity, DTP vaccine

## Abstract

The recently emerged SARS-CoV-2 causing the ongoing COVID-19 pandemic is particularly virulent in the elderly while children are largely spared. Here, we explored the potential role of cross-reactive immunity acquired from pediatric vaccinations and exposure to common human pathogens in the protection and pathology of COVID-19. To that end, we sought for peptide matches to SARS-CoV-2 (identity ≥ 80%, in at least eight residues) in the proteomes of 25 human pathogens and in vaccine antigens, and subsequently predicted their T and B cell reactivity to identify potential cross-reactive epitopes. We found that viruses subject to pediatric vaccinations do not contain cross-reactive epitopes with SARS-CoV-2, precluding that they can provide any general protection against COVID-19. Likewise, common viruses including rhinovirus, respiratory syncytial virus, influenza virus, and several herpesviruses are also poor or null sources of cross-reactive immunity to SARS-CoV-2, discarding that immunological memory against these viruses can have any general protective or pathological role in COVID-19. In contrast, we found combination vaccines for treating diphtheria, tetanus, and pertussis infectious diseases (DTP vaccine) to be significant sources of potential cross-reactive immunity to SARS-CoV-2. DTP cross-reactive epitopes with SARS-CoV-2 include numerous CD8 and CD4 T cell epitopes with broad population protection coverage and potentially neutralizing B cell epitopes in SARS-CoV-2 Spike protein. Worldwide, children receive several DTP vaccinations, including three-four doses the first year of life and one at 4–6 years of age. Moreover, a low antigenic Tdap dose is also given at ages 9–14. Thereby, children may well be protected from SARS-CoV-2 through cross-reactive immunity elicited by DTP vaccinations, supporting testing in the general population to prevent COVID-19.

## Introduction

Severe acute respiratory syndrome coronavirus 2 (SARS-CoV-2) is a new emergent virus identified in late 2019 as the etiological agent behind a mysterious outbreak of pneumonia in Wuhan, China (1, 2). The World Health Organization (WHO) later named the disease as coronavirus disease 19 (COVID-19) to account for the variety of clinical manifestations associated with SARS-CoV-2 infections (3, 4). The infectivity and pathogenicity SARS-CoV-2 have taken the world unguarded, leading to the ongoing COVID-19 pandemic.

SARS-CoV-2 infection can be asymptomatic, and the course of COVID-19 can range from mild to severe and death (5). COVID-19 cases and severity vary widely between different countries. For example, COVID-19 deaths per million reported in Spain are 606, while in Serbia, with a similar population density and geographical latitude are 30 (https://www.worldometers.info/coronavirus/). COVID-19 also varies between individuals, and medical conditions such as hypertension and obesity are risk factors (5). However, the strongest correlation factor in COVID-19 cases and severity is age. In fact, the majority of CODID-19 fatalities occur among the elderly (90% of the victims are over 70 years) while the pediatric population is largely spared (6). The large impact of COVID-19 in the geriatric population is somewhat expected, since their ability to mount immune responses to new pathogens has deteriorated (7). Yet, the virulence of the virus appears to be related to a disproportionate immune response, and indeed some patients appear to benefit from drugs with immunomodulatory properties (8). That newborns and infants, with undertrained adaptive immune systems, are safe from COVID-19 is unexpected, but telling.

Humans are not immunologically naïve, or not for long. When exposed to a new infection the host immune system preferentially engage existing cross-reactive memory B and T cells (9, 10). The promiscuity in antigen-recognition repertoires in memory B and T cells allows for quicker responses to multitude of antigens, saving energy and resources to the host. Activation of cross-reactive memory B and T cells can lead to protective immunity but also induce life-threatening immunopathology (9, 10). In this context, we reasoned that children are likely protected from SARS-CoV-2 by cross-reactive adaptive immunity elicited through vaccinations. Immunity elicited by vaccines wanes over time (11) which will leave adults more susceptible to COVID-19, and perhaps cross-reactive immunity from pathogens may in some cases protect or enhance the severity of the disease.

Adaptive immunity is driven by the recognition by T and B cells of small antigen fragments known as epitopes (12). B cell epitopes are also the targets of antibodies, and they need to be readily free and exposed to the solvent for recognition. In contrast, T cells recognize epitopes bound to major histocompatibility complex (MHC) molecules displayed in the cell surface of antigen presenting cells (12). Thereby, to test our hypothesis and identify potential sources of cross-reactive immunity to SARS-CoV-2, we conducted a systematic search for peptide matches to SARS-CoV-2 in 25 human pathogens including 18 viruses and 7 bacteria—the majority targeted by vaccinations—and in selected vaccine antigens, and predicted their T and B cell reactivity to identify cross-reactive epitopes. T cell reactivity of peptides (T cell epitopes) was predicted on the basis of their binding to MHC molecules frequently expressed in the human population using public bioinformatics tools available at IEDB (https://www.iedb.org/). B cell reactivity (B cell epitopes) was also predicted at IEDB (https://www.iedb.org/). Among the viruses without available vaccine, we considered herpes simplex virus 1 and 2, Epstein–Barr virus, human cytomegalovirus, human rhinovirus A, B, and C and respiratory syncytial virus A and B, which are all prevalent in the population. After these analyses, we found numerous cross-reactive epitopes between antigens in tetatus, diphteria, and pertussis (DTP) vaccines and SARS-CoV-2, including T cell epitopes with broad population protection coverage and potentially neutralizing B-cell epitopes. Overall, our results clearly support that cross-reactive immunity from DTP vaccines can be protecting children against SARS-CoV-2 and could protect the general population.

## Methods

### Pathogen and Vaccine Antigen Sequences

The amino acid sequences encoded by the genomes of 25 human pathogens in FASTA format were obtained from the GenBank entries indicated in **Table 1**. The amino acid sequences of antigens included in diphteria (D), tetanus (T), and pertussis cellular (wP) and acellular (aP) vaccines were obtained upon the proteomics projects PXD009289 and PXD013804 deposited in the Proteomics Identification Database (PRIDE) by the authors (13, 14) (**Table 1**). The amino acid sequence of the antigens included the meningococcal B vaccine Bexsero^®^ (MenB) was obtained upon the PRIDE proteomic project PXD011622 (15).

**Table 1:** Amino acid sequences from pathogens and vaccines considered in this study.

Pathogen	NCBI Accession	Proteins/CDS
Mumps virus (MuV)	NC_002200	8
Measles virus (MV)	NC_001498	8
Rubella Virus (RuV)	NC_001545	2
Hepatitis B virus (HBV)	NC_003977	8
Varicella Zoster Virus (VZV)	NC_001348	73
Polio Virus (PoV)	NC_002058	1
Influenza A virus (IAV)	NC_026422 … NC_026429	12
Influenza B virus (IBV)	NC_002204… NC_002211	10
Rotavirus A (RTV)	NC_011500 … NC_011510	12
Papilloma Virus (PaV)	NC_027779	6
Herpes simplex virus 1 (HSV1)	NC_001806	77
Herpes simplex virus 2 (HSV2)	NC_001798	77
Epstein–Barr virus (EBV)	NC_007605	94
human cytomegalovirus (HCMV)	NC_006273	169
human rhinovirus A (HRVA)	NC_038311	1
human rhinovirus B (HRVB)	NC_038312	1
human rhinovirus C (HRVC)	NC_009996	1
Human immunodeficiency 1 (HIV1)	NC_001802	10
bacille Calmette–Guérin (BCG)	NC_008769	3,807
*Bordetella pertussis (Bpe)*	NC_002929	3,359
*Corynebacterium diphtheriae* (Cdi)	NZ_LN831026	2,171
Clostridium tetani (Cte)	NC_004557& NC_004565	2,725
*Haemophilus influenzae* (Hin)	NC_000907	1,597
*Neisseria meningitidis* (serogroup B) (Nme)	NC_003112	1,960
*Streptococcus pneumoniae* (Spn)	NC_003098	1,821
**Vaccine Antigens**	**PRIDE PROJECT**	**Proteins/CDS**
Tetanus (T): *C. tetani*	PXD009289, PXD013804	601
Diphteria (D): *C. diphtheriae*	PXD009289, PXD013804	340
Acellular pertussis (aP): *B. pertussis*	PXD013804	25
Whole pertussis (wP): *B. pertussis*	PXD013804	1852
Meningococcal B (MenB): *N. meningitidis*	PXD011622	267

### Identification of SARS-CoV-2 Peptide Matches in Pathogens and Vaccine Antigens

Overlapping 15 mer peptides with 10 residue overlaps were generated *in silico* for each amino acid coding sequence (CDS) encoded by SARS-CoV-2 reference genome (NCBI accession NC_045512) covering their entire length. Peptides were used as queries in similarity searches using BLASTP (16) against custom made databases formatted for BLAST usage. BLAST formatted databases were generated upon FASTA files including the selected proteomes and vaccine antigens described elsewhere in *Methods*. BLAST searches were performed with default parameters, and the expected value set to 10,000. BLAST alignments were parsed using PERL scripts, and hit subject sequences consisting of eight or more residues with an identity ≥80% were selected as peptide matches with SARS-CoV-2.

### Determining Peptide Identity With Human and Microbiome Proteins

Identity between SARS-CoV-2 peptides and hits with human and microbiome proteins was obtained through local BLAST searches using an expectation value of 10,000. Human microbiome proteins were obtained from the NIH Human Microbiome Project (17) and included 6,320,906 amino acid sequences from species colonizing any/all body parts. Human proteins included 113,275 amino acid sequences and consisted of reference proteins linked to the human GRCh38 genome ensemble (https://www.ncbi.nlm.nih.gov/genome/guide/human/). Amino acid sequences of human and microbiome proteins were downloaded as FASTA files and formatted for BLAST searches.

### Prediction of T Cell Reactivity

T cell reactivity (presence of T cell epitopes) of peptides was assessed by predicting their binding to human leukocyte antigen (HLA) molecules. Peptide binding was predicted to HLA class I (HLA I) molecules HLA-A*01:01, HLA-A*02:01, HLA-A*03:01, HLA-A*11:01, HLA-A*23:01, HLA-A*24:02, HLA-B*07:02, HLA-B*08:01, HLA-B*35:01, HLA-B*40:01, HLA-B*44:02, HLA-B*44:03 using IEDB MHC I binding tool (http://tools.iedb.org/mhci/) with default recommended method through the RESTful interface. At least one of these HLA I molecules is expressed by ~95% of the world population regardless of the ethnic background as computed by the EPISOPT method (18). Models used to predict binding to HLA I molecules were the appropriated for peptides with eight and nine residues. For longer peptides, the predicted binding was obtained evaluating all encompassing 9mer peptides. A 2% percentile rank cutoff was chosen to consider that binding had occurred. Peptide binding was also predicted to HLA class II (HLA II) HLA-DR molecules encompassing the *β* chains HLA-DRB1*01:01, HLA-DRB1*03:01, HLA-DRB1*04:01, HLA-DRB1*04:05, HLA-DRB1*07:01, HLA-DRB1*08:02, HLA-DRB1*09:01, HLA-DRB1*11:01, HLA-DRB1*12:01, HLA-DRB1*13:02, HLA-DRB1*15:01 HLA-DRB3*01:01 HLA-DRB3*02:02 HLA-DRB4*01:01, HLA-DRB5*01:01 using the IEDB MHC II binding tool (http://tools.iedb.org/mhcii/) with default recommended method through the RESTful interface. A 10% percentile rank cutoff was chosen to consider that binding had occurred. HLA-DR molecules incorporate a non-polymorphic *α* chain, and the selected *β* chains are expressed by ~81% of the population as computed by the IEDB coverage tool (http://tools.iedb.org/population/) (19).

### Prediction of B Cell Reactivity

B cell or antibody reactivity (presence of B cell epitopes) of peptides was predicted using BepiPred1.0 (19) at the IEDB Analysis Resource (http://http://tools.iedb.org/bcell/). BepiPred predicts linear B cell epitopes and reports antigenicity values per residue (*a_i_*), upon which we calculated a global value of peptide antigenicity or B cell/antibody reactivity (*B*) using Eq. 1.

(1) $$B=\frac{{\sum_{i=1}^{i=l}a}_{i}}{l}$$

Where *l* is the total number of residues in the peptide. Peptides with *B* values ≥0.4 were considered as antigenic or potential B cell epitopes.

### Other Procedures

Virion surface ectodomains of SARS-CoV-2 Spike (S), membrane (M) and envelope (E) proteins were identified from UNIPROT annotation records P0DTC2, P0DTC5, and P0DTC4, respectively. PyMOL Molecular Graphics System, Version 1.8 Schrödinger, was used to map and visualize B cell epitopes on 3D structures. PDB files with three-dimensional coordinates of SARS-CoV-2 S, M, and E glyproteins were obtained from Zhong Lab (https://zhanglab.ccmb.med.umich.edu/COVID-19/) and residue relative solvent accessibility (RSA) of peptides mapping in these proteins was computed using NACCESS (20). We used the non-parametric Kruskal–Wallis test to compare COVID-19 cases and death rates in European countries, including Asian countries from former URSS, with regard to the inclusion of DTaP or DTwP vaccines. Vaccination data was obtained from (https://apps.who.int/immunization_monitoring/) and death rates from (https://www.worldometers.info/coronavirus/).

## Results and Discussion

### Identification Cross-Reactive Immunity to SARS-CoV-2

Adaptive immunity is mediated by B and T cells which recognize small fragments, epitopes in the target antigens. Thereby, to identify sources of potential cross-reactive immunity to SARS-CoV-2, we devised a method to search for peptide matches between SARS-CoV-2 and sources of interest (details in *Methods*). Briefly, we partitioned the entire SARS-CoV-2 into 15 mer peptides with 10 residue overlaps and used them as query in modified BLAST searches against subject proteomes. We then selected ungapped subject hits with eight or more residues and ≥80% identity to SARS-CoV-2 as potential sources of cross-reactive immunity. We next predicted B cell and T cell reactivity of peptide hits using relevant epitope prediction methods (details in *Methods*). T cells can be divided into CD4 and CD8 T cells which recognize peptides displayed in the cell surface of antigen presenting cells bound to class I and class II MHC molecules, respectively. Therefore, we anticipated CD4 and CD8 T cell epitopes by predicting peptide binding to class I and class II human MHC molecules (in human known as HLA molecules for Human Leukocyte Antigens). For realization of T cell cross-reactivity, both peptide hits and equivalent SARS-CoV-2 peptides have to bind to the same class I and/or class II HLA molecules. Peptide hits with B cell epitope scores ≥0.4 were considered to be cross-reactive for B cell/antibody mediated immunity.

We first sought for cross-reactive immunity to SARS-CoV-2 in the proteomes of 25 human pathogens, including 18 viruses not related to SARS-CoV-2 and seven bacteria. Cross-reactive heterologous immunity is not limited to closely related pathogens, and in fact, it can occur between unrelated viruses (21) and between bacteria and viruses (22). As we age, there is a reduction in naive T cells and T cell repertoire diversity (23–25), likely prevailing memory T cells specific for pathogens causing common and/or persistent infections as shown for human cytomegalovirus (26). Therefore, in this study we considered viruses causing common, persistent and/or recurrent infections, including influenza (A and B), various herpesviruses (Epstein–Barr Virus, human cytomegalovirus and herpes simplex virus 1 and 2), human rhinovirus (HRV) A, B and C, human respiratory syncytial virus (RSV) A and B and human immunodeficiency virus 1 (HIV1). We also considered viruses targeted by vaccinations including poliovirus (PoV), mumps virus (MuV), rubella virus (RuV), papilloma virus (PaV), and rotavirus A (RTVA). Among the bacteria we considered bacille Calmette–Guérin (BCG), *Bordetella pertussis* (*Bpe*), *Corynebacterium diphtheriae* (*Cdi*), *Clostridium tetani* (Cte), *Haemophilus influenzae* (Hin), *Neisseria meningitidis* (serogroup B) and *Streptococcus pneumoniae* (Spn), which are all targeted by vaccinations. We found 595 unique peptides in the analyzed proteomes matching 551 peptides from SARS-CoV-2, indicating that the potential cross-reactive epitopes with SARS-CoV-2 differ for each pathogen. The complete data is provided in **Supplementary Dataset 1**, and in **Table 2** we summarize the results. Overall, the number of SARS-CoV-2 peptide matches as well as that of potential cross-reactive B and T cell epitopes correlated with the size of the proteomes (**Table 2**). We cannot discard that some cross-reactive epitopes may be smaller and/or share less identity. In addition, there might be some cross-reactive epitopes rising from pathogen variants that we could not detect as we used single reference proteomes. We used state-of-the-art bioinformatics tools to predict cross-reactive T and B cell epitopes, but if we had used alternative prediction methods the cross-reactive T and B cell epitopes may be somewhat different. Overall, the cross-reactive epitopes detected through this approach will need experimental confirmation.

**Table 2:** Summary of potential cross-reactivity immunity to SARS-CoV-2 from selected pathogens.

	SARS-Peptide Hits^(1)^	B^(2)^	CD8 T^(3)^	CD4 T^(4)^	Vaccine ^(5)^
Mumps virus (MuV)	2	1	0	0	Y
Measles virus (MeV)	0	0	0	0	Y
Rubella Virus (RuV)	0	0	0	0	Y
Hepatitis B virus (HBV)	1	1	0	0	Y
Varicella Zoster Virus (VZV)	4	1	2	0	Y
Polio Virus (PoV)	0	0	0	0	Y
Papilloma Virus (PaV)	2	1	1	1	Y
Rotavirus A (RTVA)	0	0	0	0	Y
Influenza A virus (IAV)	0	0	0	0	Y
Influenza B virus (IBV)	0	0	0	0	Y
Herpes simplex virus 1 (HSV1)	0	0	0	0	N
Herpes simplex virus 2 (HSV2)	2	1	0	0	N
Epstein–Barr virus (EBV)	9	3	2	1	N
human cytomegalovirus (HCMV)	1	0	0	0	N
human rhinovirus A (HRVA)	1	1	0	0	N
human rhinovirus B (HRVB)	0	0	0	0	N
human rhinovirus C (HRVC)	0	0	0	0	N
Human Immunodeficiency 1 (HIV1)	0	0	0	0	N
Bacille Calmette-Guérin (BCG)	120	41	21	11	Y
Bordetella pertussis (Bpe)	111	36	18	12	Y
Corynebacterium diphtheriae (Cdi)	83	33	14	5	Y
Clostridium tetani (Cte)	85	34	17	7	Y
Haemophilus influenzae (Hin)	53	16	6	5	Y
Neisseria meningitides (Nme)	60	23	9	3	Y
Streptococcus pneumoniae (Spn)	61	19	13	6	Y

^1^Number of peptides in the relevant pathogen with ≥8 residues and identity ≥80% with SARS-CoV-2. ^2^Peptide hits with B cell reactivity ≥0.4 (predicted as cross-reactive B cell epitopes). ^3^Peptides predicted as cross-reactive CD8 T cell epitopes. ^4^Peptides predicted as cross-reactive CD4 T cell epitopes. ^5^Whether these pathogens are subject to vaccinations. Whole dataset available in **Supplementary Dataset 1**.

### Cross-Reactive Immunity to SARS-CoV-2 From Prevalent Viruses and Viruses Targeted by Vaccinations

A recent study has detected SARS-CoV-2 reactive CD4 T cells in unexposed individuals, suggesting cross-reactive T cell recognition between circulating common cold coronaviruses and SARS-CoV-2 (27). T cell cross-reactivity between SARS-CoV-2 and other circulating coronaviruses is expected as they share high sequence similarity. However, whether infection by common cold coronaviruses leads to such cross-reactivity remains to be confirmed. In fact, common cold coronaviruses are responsible for less than 10% of all common colds of viral etiology (28). In the virus included in this study, there are only 22 SARS-CoV-2 peptide matches, including nine peptides with predicted B cell/antibody reactivity and five with predicted T cell cross-reactivity (**Table 2**). Two SARS-CoV-2 peptides in the Spike protein, the target of protective neutralizing antibodies (29), are present in MuV and VZV (**Table 3**). However, these two peptides have no predicted B cell reactivity, and more importantly do not map in accessible regions (data not shown). Of the 5 T cell cross-reactive epitopes, two are present in EBV; one of them (APSASAPF) is restricted by two HLA I molecules (B*07:02 and B*35:01) and the other (DLLLDASVEI) by one HLA I (A*02:01) and two HLAII (HLA-DRB1*03:01 and HLA-DRB3*01:01) molecules. No vaccine is available for EBV, and these cross-reactive epitopes may be drivers of protective or pathological immune responses in the individual expressing the noted HLA molecules. Such a role could be unraveled through functional recall assays (*e.g.* by ELISPOT assays) aimed to identify peptide-specific T cell responses in EBV^+^ and EBV^−^ HLA type subjects with a history of SARS-CoV-2 infection, including asymptomatic and convalescents from severe disease. The other three potential cross-reactive T cell epitopes are found in two viruses targeted by vaccinations (**Table 3**). There are two CD8 T cell epitopes in VZV, one restricted by B*35:01 and another by B*08:01 and additional epitope in PaV that can be presented by class I (A*02:01) and class II (DRB1*01:01, HLA-DRB1*03:01, HLA-DRB1*09:01) HLA molecules (**Table 3**). PaV vaccines consist of recombinant L1 proteins (30) while the potentially cross-reactive PaV epitope maps in the E1 protein (**Table 3**). Thereby, PaV infection but not the vaccine can elicit T cell cross-reactivity to SARS-CoV-2. Overall, we can conclude that the selected viruses and particularly those targeted by vaccinations are poor sources of immune cross-reactivity to SARS-CoV-2 (**Tables 2**, **3**). Thereby, we can discard that current pediatric vaccinations for viral diseases protect against SARS-CoV-2 infection, as recently suggested for RuV vaccine (31). Such suggestion was based on the distant homology observed between SARS-CoV-2 NS3P and Rubella virus p150 proteins, but not a single peptide is shared between SARS-CoV-2 and RuV.

**Table 3:** Potential B and T cell cross-reactive epitopes in selected viral pathogens.

ACN|ANTIGEN|^(1)^SARS_CoV-2	SEQUENCE^(2)^	HIT ACN^(3)^|VIRUS|	HIT SEQ^(4)^	ID (%)^(5)^	B^(6)^	CD8 T^(7)^	CD4 T^(8)^
YP_009724390 |SPIKE-269-283|	YLQPRTFLLKYNENG	NP_054707.1 |MuV|	LLRYNENG	87.5	0.0	NP	NP
YP_009725299 |NSP3-1805-1819|	NVSLDNVLSTFISAA	NP_054711.1 |MuV|	LSTSISAA	87.5	0.8	NP	NP
YP_009724390 |SPIKE-989-1003|	AEVQIDRLITGRLQS	NP_040162.2 |VZV|	LITGHLQS	87.5	−0.5	NP	NP
YP_009724397.2 |NP-213-227|	NGGDAALALLLLDRL	NP_040149.1 |VZV|	GYADLALLLL	80	−1.7	HLA-B*35:01	NP
YP_009725299 |NSP3-737-751|	NLKTLLSLREVRTIK	NP_040145.1 |VZV|	NLKTLLKL	87.5	−1.5	HLA-B*08:01	NP
YP_009725304 |NSP8-69-83|	QMYKQARSEDKRAKV	NP_040156.1 |VZV|	SEDKLAKV	87.5	1.0	NP	NP
YP_009725297 |LP-9-23|	NEKTHVQLSLPVLQV	YP_009163893.1 |PaV|	VQLSLPPLLV	80	−0.7	HLA-A*02:01	HLA-DRB1*01:01, HLA-DRB1*03:01, HLA-DRB1*09:01
YP_009725308 |HEL-277-291|	YSTLQGPPGTGKSHF	YP_009163893.1 |PaV|	GPPDTGKSLF	80	2.4	NP	NP
YP_009725308 |HEL-277-291|	YSTLQGPPGTGKSHF	YP_009505608.1 |HRVA|	IHGPPGTGKS	80	2.1	NP	NP
YP_009724391 |ORF3a-245-259|	QIHTIDGSSGVVNPV	YP_009173870.1 |HBV|	SSGTVNPV	87.5	1.1	NP	NP
YP_009725299 |NSP3-1493-1507|	ISSFKWDLTAFGLVA	YP_009137182.1 |HSV2|	LTAAGLVA	87.5	−0.5	NP	NP
YP_009725298 |NSP2-141-155|	MKCDHCGETSWQTGD	YP_009137214.1 |HSV2|	EPSWQTGD	87.5	1.6	NP	NP
YP_009725303 |NSP7-9-23|	TSVVLLSVLQQLRVE	YP_081506.1 |HCMV|	LLSALQQL	87.5	−1.2	NP	NP
YP_009724397 |NP-301-315|	WPQIAQFAPSASAFF	YP_401663.1 |EBV|	APSASAPF	87.5	1.4	HLA-B*07:02, HLA-B*35:01	NP
YP_009725299 |NSP3-1289-1303|	IKKPNELSRVLGLKT	YP_401644.1 |EBV|	ELSPVLGL	87.5	−0.4	NP	NP
YP_009725302 |NSP6-229-243|	CLLNRYFRLTLGVYD	YP_401712.1 |EBV|	FRLTGGVY	87.5	−0.3	NP	NP
YP_009725306 |NSP10-45-59|	LCTHTGTGQAITVTP	YP_401662.1 |EBV|	TSQAITVT	87.5	0.5	NP	NP
YP_009725307 |POL-21-35|	PCGTGTSTDVVYRAF	YP_401694.1 |EBV|	GTGTSTGV	87.5	1.5	NP	NP
YP_009725307 |POL-257-271|	VDTDLTKPYIKWDLL	YP_401667.1 |EBV|	TVPYIKWD	87.5	−0.1	NP	NP
YP_009725308| HEL-241-255	VPQEHYVRITGLYPT	YP_401712.1 |EBV|	VRITGLIP	87.5	−0.8	NP	NP
YP_009725310 |RNASE-249-263|	HLLIGLAKRFKESPF	YP_401688.1 |EBV|	LKKRFKESYF	80	−0.3	NP	NP
YP_009725310 |RNASE-293-307|	SVIDLLLDDFVEIIK	YP_401687.1 |EBV|	DLLLDASVEI	80	−0.8	HLA-A*02:01	HLA-DRB1*03:01, HLA-DRB3*01:01

^1^Accession and antigen source of SARS-CoV-2 peptide. ^2^Sequence of peptide used in BLAST searches. ^3^Accession and source virus of BLAST hit. ^4^Sequence of peptide hit. ^5^Percentage of identity between peptide hit and equivalent SARS-CoV-2 peptide. ^6^B cell/Antibody reactivity (peptide hits with B ≥ 0.4 were considered as cross-reactive B cell epitopes). ^7^CD8 T cell cross-reactivity given by the HLA I molecules predicted to bind both the peptide hit and the equivalent SARS-CoV-2 peptide. ^8^CD4 T cell cross-reactivity given by the HLA II molecules predicted to bind both the peptide hit and the equivalent SARS-CoV-2 peptide. NP, None Predicted.

### Cross-Reactive Immunity to SARS-CoV-2 From Bacteria Targeted by Vaccinations

In contrast to viruses, the selected bacterial proteomes are considerable sources of cross-reactive immunity to SARS-CoV-2, correlating with their large proteomes (**Table 2**). However, the potential immune cross-reactivity rising from these bacteria shall be considered relevant. The match between SARS-CoV-2 and bacteria peptidomes is quite private; only 28 SARS-CoV-2 peptides out of 535 have a match with more than one bacterial proteome. Moreover, 88% of SARS-CoV-2 peptide matches with bacteria are absent in human or have an identity under that with bacteria (see **Supplementary Dataset 1**). The majority (68%) of cross-reactive SARS-CoV-2 peptides with bacteria do also match the human microbiome (44% with higher identity) (see **Supplementary Dataset 1**). This finding might look trivial, but it is actually very interesting. It has been noted that maintaining a diverse microbiota is essential for a competent immunity system (32, 33). Loss of microbiota diversity is associated with many diseases; it occurs in the elderly and immunocompromised subjects, and it can increase susceptibility to viral infections (34). How a diverse microbiota helps to fight viral infections is unclear, but it could be by increasing the chance for cross-reactive immunity. Interestingly, it has been shown that epitope similarity to the microbiota can enhance the immunogenicity of disease-associated antigenic epitopes (35).

The proteomes of BCG, *B. pertussis*, *C. diphtheriae*, *C. tetani*, *H. influenzae*, *N. meningitidis* and *S. pneumoniae*, contain numerous potentially cross-reactive epitopes, and make us wonder whether vaccination against these bacteria could elicit protective heterologous immunity against SARS-CoV-2 (**Table 2**). Such possibility has already been proposed for BCG (36–38) but through a different mechanism. BCG is a live attenuated strain of *Mycobacterium bovis* used to prevent tuberculosis (TB) (39), which is known to confer heterologous protective immunity to unrelated pathogens, including viruses, by inducing trained immunity (40, 41). Heterologous trained immunity is antigen non-specific and involves functional reprogramming of innate immune cells (42). It was the observation that there are less COVID-19 cases and fatalities in countries implementing BCG vaccination that led to postulate that trained immunity resulting from BCG vaccination could confer some protection against SARS-CoV-2 (36–38). Although we fully acknowledge the concept of trained immunity, our results also support that, if any, long-term protection against SARS-CoV-2 induced by BCG is most likely due to the presence of cross-reactive epitopes. We, however, find questionable that current BCG vaccinations are responsible for long-term protection against SARS-CoV-2.

BCG is only given once at birth, and long-term protection against any pathogen generally requires repeated antigen re-exposure to maintain memory T and B cells (43). Moreover, BCG vaccination is only included in some countries, while children and teenagers are largely unaffected by SARS-CoV-2 worldwide. In line with these arguments, a recent epidemiological study has discarded that BCG vaccination in childhood has a protective effect against COVID-19 in adulthood (44). Therefore, we next turned to analyze if immune cross-reactivity to SARS-CoV-2 from vaccines to *C. diphtheriae, B. pertusis, C. tetani, H. influenzae, N. meningitidis and S. pneumoniae* is what keeps children and young people safe from COVID-19.

### Cross-Reactive Immunity to SARS-CoV-2 From Bacterial Vaccines: DTP and MenB

Pediatric vaccines for bacterial disease differ widely, and we first investigated their composition. Vaccines for *H. influenzae* (Hib), *S. pneumoniae* (PCV), and *N. meningitides serotypes* A, C, W, and Y (MenC and MenACWY) consist of bacteria cell wall polysaccharides, alone or conjugated with tetatus and/or diphteria toxoids (45–48). In contrast, vaccines for *N. meningitides* serotype B (MenB) consist of surface proteins from *N. meningitides* (15). Diphteria (D) and tetanus (T) vaccines contain inactivated toxins produced by the bacteria (toxoids) but in addition include a large array of proteins as shown by proteomics studies, which can be immunogenic (14, 49). D and T antigens are generally combined with pertussis (P) antigens as a combination DTP vaccine, which is available in two main formulations: DTaP and DTwP. DTaP contains selected P antigens without cells (acellular pertusis, aP), and DTwP includes inactivated whole pertussis bacteria (wP) (50).

After considering the nature of bacterial vaccines, we discarded conjugate and polysaccharides vaccines and sought for potential sources of cross-reactive immunity in antigens identified by proteomics in DTa/wP and MenB vaccines. D and T antigens can vary between different vaccines, and here we considered the sum of all those reported elsewhere in (13, 14, 49). Our results, summarized in **Table 4**, indicate that combination DTP vaccines are significant sources of B and T cell cross-reactivity to SARS-CoV-2, while such cross-reactivity is small from MenB vaccine. In DTaP, P antigens do not contribute to increase the cross-reactive immunity provided by D and T antigens, but in DTwP, wP provide as much cross-reactive immunity as D and T antigens together (**Table 4**).

**Table 4:** Summary of potential cross-reactive immunity to SARS-CoV-2 from selected vaccine antigens.

Vaccine Antigens	Proteins	SARS-Peptide Hits^(1)^	B^(2)^	CD8 T^(3)^	CD4 T^(4)^
D	340	24	6	5	2
T	601	28	13	8	2
aP	25	3	1	1	0
wP	1,852	67	23	10	5
MenB	267	10	3	2	1

^1^Number of peptides in the relevant antigens with ≥8 residues and identity ≥80% with SARS-CoV-2. ^2^Peptide hits with B cell reactivity ≥0.4 (predicted as cross-reactive B cell epitopes). ^3^Peptides predicted as cross-reactive CD8 T cell epitopes. ^4^Peptides predicted as cross-reactive CD4 T cell epitopes. Whole dataset available in **Supplementary Dataset 2**.

It is interesting to note that combination DTP vaccines, particularly DTwP, appear to be good inducers of cross-reactive CD8 T cells, which are key to clear viral infections by killing infected cells. The elicitation of virus cross-reactive CD8 T cells with bacterial antigens can occur through cross-presentation. This process enables antigen presenting cells to display endocytosed antigens in the context of HLA I molecules and prime CD8 T cells with anti-viral activity (51). We actually found more cross-reactive CD8 T cell epitopes than CD4 T cell epitopes (**Table 5**). However, this imbalance can be due to the fact that peptides with CD8 T cell epitopes can be shorter than those with CD4 T cell epitopes. In any case, the number of CD8 and CD4 T cell epitopes does not necessarily match with the magnitude of the CD8 and CD4 T cell response to SARS-CoV-2. In fact, SARS-CoV-2 specific CD4 T cell responses appear to be dominant when stimulating peripheral mononuclear blood cells with overlapping peptides (52). Interestingly, some cross-reactive T cell epitopes in DTP and MenB vaccines can be presented by HLA I and HLA II molecules, which will enable them to elicit both, CD8 and CD4 T cell responses. Moreover, many of the cross-reactive CD8 and CD4 T cell epitopes can be presented by various HLA I and HLA II molecules, respectively. T cell epitopes are only immunogenic in those individual expressing the HLA molecules that restrict the responses. With our data, we computed that the percentage of the world population that will have cross-reactive CD8 T cell responses to SARS-CoV-2 from MenB, DTaP, and DTwP vaccinations will be 46.54, 65.59, and 85.79%, respectively. Moreover, the percentage of the world population that will have cross-reactive CD8 and CD4 T cells to SARS-CoV-2 from MenB, DTaP, and DTwP vaccines will be 70.9, 77.47, and 94.67%, respectively. For populations expressing low frequency HLA molecules not included in this study, a more specific analysis considering such molecules will be required to uncover the relevant cross-reactive T cell epitopes and their coverage in that population. Overall, however, we can confirm that combination DTP vaccines, and particularly DTwP, would be able to elicit cross-reactive T cell responses against SARS-CoV-2 in most individuals.

**Table 5:** Potential cross-reactive T cell epitopes from DTP and MenB vaccine antigens.

SARS-CoV-2 Antigen^(1)^	SARS-CoV-2 query sequence^(2)^	HIT ACN^(3)^	Vaccine^(4)^	ID %^(5)^	HIT SEQ^(6)^	CD8 T^(7)^	CD4 T^(8)^
YP_009724394|ORF6-33-47	INLIIKNLSKSLTEN	FTSA_ NEIMB	MenB	81.8	NLITKDLSKSL	HLA-A*03:01, HLA-A*11:01, HLA-B*07:02, HLA-B*08:01	HLA-DRB1*03:01, HLA-DRB1*07:01, HLA-DRB1*08:02, HLA-DRB1*12:01, HLA-DRB1*13:02, HLA-DRB3*01:01
YP_009724392|ENV-45-59	NIVNVSLVKPSFYVY	Q9K1E8_ NEIMB	MenB	80.0	VNVSLVDLSF	HLA-A*23:01, HLA-B*35:01	NP
YP_009725311|2ORMT-89-103	LPTGTLLVDSDLNDF	Q6NGY6_CORDI	D (DTaP/wP)	80.0	LLVDADLTDF	HLA-A*01:01, HLA-B*35:01	HLA-DRB1*03:01, HLA-DRB3*01:01
YP_009725298|NSP2-93-107	FVFPLNSIIKTIQPR	Q6NJR6_ CORDI	D (DTaP/wP)	88.9	SIIKTIQDR	HLA-A*03:01, HLA-A*11:01	HLA-DRB1*03:01, HLA-DRB4*01:01
YP_009724390|SPIKE-161-175	SSANNCTFEYVSQPF	Q6NIW5_ CORDI	D (DTaP/wP)	87.5	FEYVSQNF	HLA-B*40:01, HLA-B*44:02, HLA-B*44:03	NP
YP_009725309|35EXON-1-15	AENVTGLFKDCSKVI	Q6NFL4_ CORDI	D (DTaP/wP)	87.5	AENVTGFF	HLA-B*40:01, HLA-B*44:02, HLA-B*44:03	NP
YP_009725307|POL-173-187	RVYANLGERVRQALL	SYI_ CORDI	D (DTaP/wP)	87.5	EGVRQALL	HLA-B*08:01	NP
YP_009725309|35EXON-285-299	CFVKRVDWTIEYPII	Q899B2_ CLOTE	T (DTaP/wP)	80.0	LKRVDWDIEY	HLA-B*35:01, HLA-B*44:02, HLA-B*44:03	HLA-DRB3*01:01
YP_009725298|NSP2-69-83	YELQTPFEIKLAKKF	Q894R8_ CLOTE	T (DTaP/wP)	87.5	EIKKAKKF	HLA-B*08:01, HLA-B*44:02, HLA-B*44:03	NP
YP_009725297|LP-85-99	MVELVAELEGIQYGR	Q891V6_ CLOTE	T (DTaP/wP)	80.0	LVAILEGIRY	HLA-A*01:01, HLA-B*35:01	NP
YP_009725309|35EXON-289-303	RVDWTIEYPIIGDEL	Q899B2_ CLOTE	T (DTaP/wP)	87.5	RVDWDIEY	HLA-A*01:01, HLA-B*35:01	NP
YP_009725308|HEL-277-291	YSTLQGPPGTGKSHF	Q891B9_ CLOTE	T (DTaP/wP)	80.0	LVGPPGTGKT	HLA-A*03:01, HLA-A*11:01	NP
YP_009724390|SPIKE-1061-1075	VFLHVTYVPAQEKNF	Q899V7_ CLOTE	T (DTaP/wP)	80.0	TNVHAQEKNF	HLA-B*35:01	NP
YP_009725308|HEL-277-291	YSTLQGPPGTGKSHF	Q895L6 _CLOTE	T (DTaP/wP)	80.0	LYGPPGTGKT	HLA-A*03:01	NP
YP_009725308|HEL-133-147	FAAETLKATEETFKL	Q899L8_ CLOTE	T (DTaP/wP)	88.9	KATEETVKL	HLA-B*35:01	NP
YP_009725307|POL-137-151	GNCDTLKEILVTYNC	Q899S9_ CLOTE	T (DTaP/wP)	87.5	LKEILVNYN	NP	HLA-DRB1*04:05, HLA-DRB1*12:01
YP_009725308|HEL-561-575	FNVAITRAKVGILCI	Q7VX10_BORPE	wP/aP (DTaP/wP)	87.5	TGAKVGIL	HLA-B*08:01	NP
YP_009724390|SPIKE-509-523	RVVVLSFELLHAPAT	Q7VSF2_ BORPE	wP (DTwP)	80.0	SFELAEAPAT	HLA-B*40:01	HLA-DRB1*01:01, HLA-DRB1*04:01, HLA-DRB1*04:05, HLA-DRB1*09:01
YP_009725299|NSP3-857-871	YLATALLTLQQIELK	Q7VWJ0_ BORPE	wP (DTwP)	80.0	ALLTLQQLHL	HLA-A*02:01	HLA-DRB4*01:01
YP_009724397|NP-217-231	AALALLLLDRLNQLE	Q7VSK8_ BORPE	wP (DTwP)	80.0	ARLALLPLDR	HLA-A*03:01, HLA-A*11:01	NP
YP_009725298|NSP2-69-83	YELQTPFEIKLAKKF	Q7VVM6 BORPE	wP (DTwP)	87.5	SPFEIKLA	HLA-B*07:02, HLA-B*35:01	NP
YP_009724397|NP-209-223	RMAGNGGDAALALLL	Q7VUJ1_ _BORPE	wP (DTwP)	80.0	QAGQGGDAAL	HLA-B*07:02	NP
YP_009724390|SPIKE-449-463	YNYLYRLFRKSNLKP	Q7VZJ7 _BORPE	wP (DTwP)	87.5	RLFRKSLL	HLA-B*08:01	NP
YP_009725302|NSP6-97-111	WLDMVDTSLSGFKLK	Q7VXB3_ BORPE	wP (DTwP)	87.5	SLSGGKLK	HLA-A*03:01	NP
YP_009725299|NSP3-249-263	HGGGVAGALNKATNN	Q7VVD1_ BORPE	wP (DTwP)	80.0	GGVAGALFAA	HLA-A*02:01	NP
YP_009725299|NSP3-521-535	VEEAKTVLKKCKSAF	RL19_ BORPE	wP (DTwP)	80.0	KLVLKKAKSA	HLA-B*08:01	NP
YP_009724391.1|ORF3a-41-55	LPFGWLIVGVALLAV	Q7VZ84_ BORPE	wP (DTwP)	88.9	LIVGLALLA	NP	HLA-DRB1*01:01, HLA-DRB1*08:02, HLA-DRB1*15:01
YP_009724392.1|ENV-45-59	NIVNVSLVKPSFYVY	Q7VS52_ BORPE	wP (DTwP)	80.0	VVIVSLVKPS	NP	HLA-DRB1*03:01, HLA-DRB1*08:02
YP_009725307|POL-749-763	LRKHFSMMILSDDAV	PTLC_ BORPE	wP (DTwP)	80.0	HFSMMILGAD	NP	HLA-DRB1*04:05, HLA-DRB4*01:01

^1^Accession and antigen source of SARS-CoV-2 peptide. ^2^Sequence of peptide used in BLAST searches. ^3^Accession of BLAST hit bacterial antigen. ^4^Vaccine incorporating antigen. All vaccines with D or T antigens are labeled as DTa/wP vaccine formulations. ^5^Percentage of identity of peptide hit to equivalent SARS-CoV-2 peptide. ^6^Sequence of bacterial peptide hit. ^7^CD8 T cell cross-reactivity given by the HLA I molecules predicted to bind both the hit peptide and the equivalent SARS-CoV-2 peptide. ^8^CD4 T cell cross-reactivity given by the HLA II molecules predicted to bind both the peptide hit and the equivalent SARS-CoV-2 peptide. NP, None Predicted.

Elicitation of effective cross-reactive T cell responses to SARS-CoV-2, particularly CD8 T cell responses, would allow the host to clear the virus quickly and exhibit milder courses of COVID-19. However, children appear to be largely unaffected by the virus, which is consistent with having protective antibody immunity blocking and limiting viral infection. Interestingly, such protective immunity could also result from cross-reactive antibodies elicited by combination DTP vaccines. Thus, we found 16 SARS-CoV-2 peptide matches with DTP antigens, mapping in ectodomains of virion surface antigens and of those, 14 are in SARS-CoV-2 Spike protein (**Table 6**). None of the SARS-CoV-2 peptide matches mapping in virion surface antigens are from aP antigens, while 11 are from wP, highlighting the superiority of DTwP vaccines in eliciting potentially protective cross-reactive immunity to SARS-CoV-2. It is worth noting that some SARS-CoV-2 peptide matches such as FEYVSQPF are not predicted as B cell epitopes (*B* < 0.4) but yet could be readily recognized by antibodies as shown by the computed solvent accessibilities (**Table 6**). B cell epitope predictions methods are in general quite unreliable, and actual solvent accessibilities values are better measures of potential B cell reactivity.

**Table 6:** Potential cross-reactive B cell epitopes from DTP and MenB vaccine antigens.

SARS-CoV-2 Surface Antigen^(1)^	HIT ACN^(2)^	Vaccine^(3)^	SARS-CoV-2 Peptide^(4)^	HIT PEP^(5)^	ID %^(6)^	Virion Surface^(7)^	B^(8)^	Acc (%)^(9)^
YP_009724390 SPIKE-1-15	ATPG_ BORPE	wP (DTwP)	VFLVLLPL	VFLRLLPL	87.5	N	−2.4	NA
YP_009724390 SPIKE-161-175	Q6NIW5_ CORDI	D (DTa/wP)	FEYVSQPF	FEYVSQNF	87.5	Y	−0.3	62.50
YP_009724390 SPIKE-233-247	Q7VZN3_ BORPE	wP (DTwP)	QTLLALHR	QTLLATHR	87.5	Y	−0.7	9.20
YP_009724390 SPIKE-253-267	Q7VVR9_ BORPE	wP (DTwP)	SSSGWTAGAAA	QSSGWTAGADA	81.8	Y	1.3	19.10
YP_009724390 SPIKE-449-463	Q7VZJ7_ BORPE	wP (DTwP)	RLFRKSNL	RLFRKSLL	87.5	Y	−1.4	66.00
YP_009724390 SPIKE-509-523	Q7VSF2_ BORPE	wP (DTwP)	SFELLHAPAT	SFELAEAPAT	80.0	Y	0.6	44.71
YP_009724390 SPIKE-509-523	SMG_ BORPE	wP (DTwP)	ELLHAPAT	ELAHAPAT	87.5	Y	0.6	51.73
YP_009724390 SPIKE-701-715	Q7VX63_ BORPE	wP (DTwP)	SVAYSNNS	SVAYSNNS	100.0	Y	0.7	84.18
YP_009724390 SPIKE-893-907	Q7VSI7_ BORPE	wP (DTwP)	IPFAMQMA	IPFTMQMA	87.5	Y	−0.7	48.84
YP_009724390 SPIKE-953-967	Q6NG46 _CORDI	D (DTa/wP)	ALNTLVKQ	ALNGLVKQ	87.5	Y	−0.6	32.18
YP_00972439 SPIKE-989-1003	Q7VSP7_ BORPE	wP (DTwP)	DRLITGRL	DRLIEGRL	87.5	Y	−0.5	36.80
YP_009724390 SPIKE-1061-1075	Q899V7_ CLOTE	wP (DTa/wP)	TYVPAQEKNF	TNVHAQEKNF	80.0	Y	1.1	26.87
YP_009724390 SPIKE-1089-1103	CARA_ CORDI	D (DTa/wP)	EGVFVSNG	DGVFVSNG	87.5	Y	−0.1	22.30
YP_009724390 SPIKE-1145-1159	Q898W3_ CLOTE	T (DTa/wP)	EELDKYFK	EKLDKYFK	87.5	Y	−0.2	35.95
YP_009724390 SPIKE-1221-1235	SYFB_ BORPE	wP (DTwP)	IAGLIAIV	IAGLVAIV	87.5	Y	−1.5	2.70
YP_009724393 MP-5-19	CH60_ BORPE	wP (DTwP)	TVEELKKL	AVEELKKL	87.5	Y	−0.3	45.90
YP_009724393 MP-5-19	Q893U5_ CLOTE	T (DTa/wP)	VEELKKLL	VEELKKEL	87.5	Y	−0.1	23.63
YP_009724393 MP-157-171	Q897F9_ CLOTE	T (DTa/wP)	IKDLPKEI	IKDNPKEI	87.5	N	1.3	NA
YP_009724392 ENV-9-23	Q7VSN6_ BORPE	wP (DTwP)	SVLLFLAF	SVLKFLAF	87.5	N	−2.0	NA
YP_009724392 ENV-45-59	Q7VS52_ BORPE	wP (DTwP)	IVNVSLVKPS	VVIVSLVKPS	80.0	N	−1.0	NA
YP_009724392 ENV-45-59	GLPK1_ CLOTE	T (DTa/wP)	VKPSFYVY	VKPSSYVY	87.5	N	1.4	NA
YP_009724392 ENV-45-59	Q9K1E8_NEIMB	MenB	VNVSLVKPSF	VNVSLVDLSF	80.0	N	0.0	NA

^1^SARS-CoV-2 surface antigen. ^2^Accession of antigens with potential cross-reactivity. ^3^Vaccine antigen. For D, T, aP, and wP antigens we show in parenthesis the vaccine incorporating that antigen. D and T antigens are noted to be in DTaP and DTwP vaccines (DTa/wP) ^4^SARS-CoV-2 peptide sequence. ^5^Peptide hit sequence with identity to SARS-CoV-2 peptide.^6^Sequence Identity (%) between SARS-CoV-2 and hit peptides. ^7^Whether the peptide map in a region of the antigen located at the virion surface. ^8^B cell/Antibody reactivity (peptide hits with B ≥ 0.4 are predicted as cross-reactive B cell epitopes). ^9^Solvent accessibility of SARS-CoV-2 peptide in percentage computed upon residue relative solvent accessibilities from the relevant 3D-structures (details in Methods). NA: Not Applicable.

**Figure 1:**
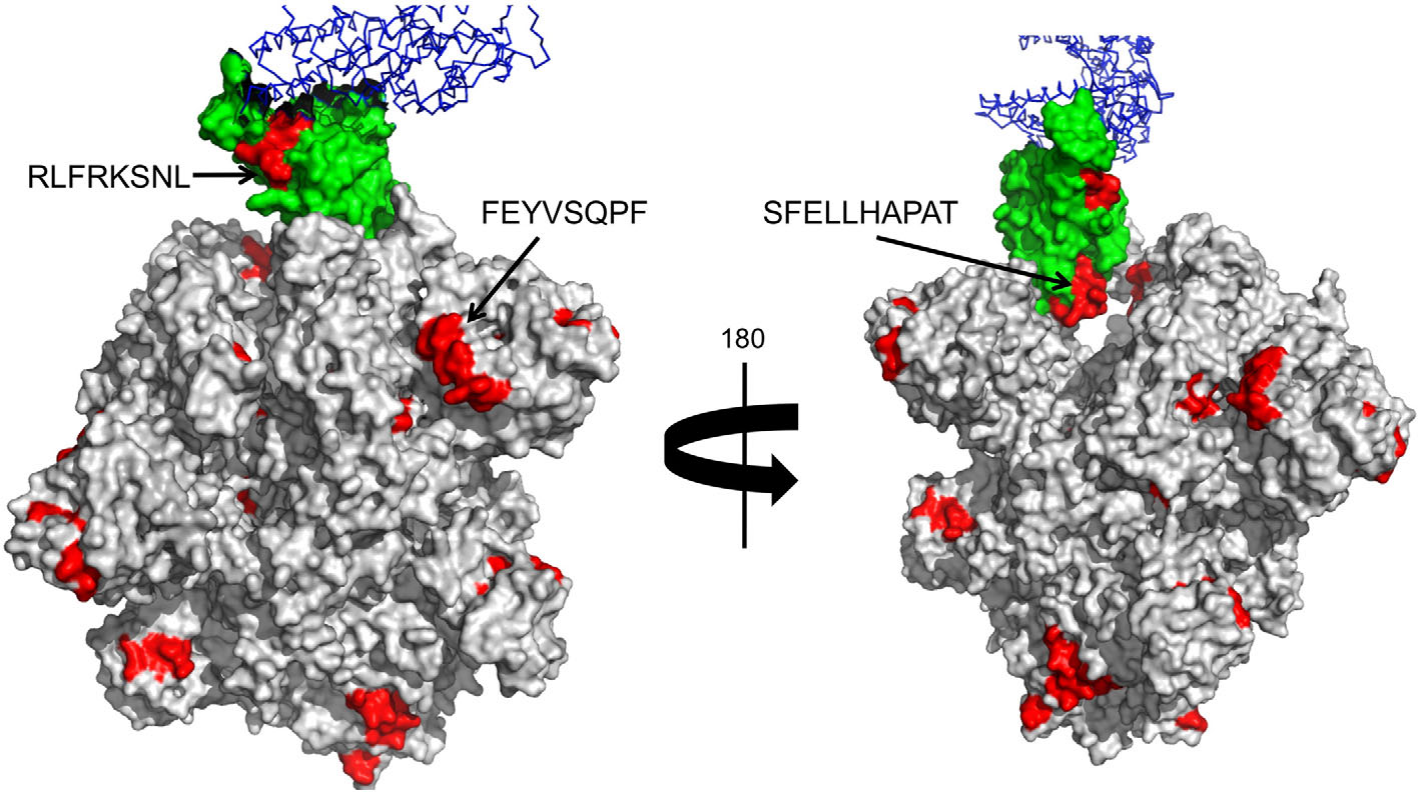
Cross-reactive B cell epitopes tagging SARS-CoV-2 Spike protein. Panel shows a surface rendering of the tertiary structure of the trimeric SARS-CoV-2 Spike protein in the open state with cross-reactive B cell epitopes in red. The Spike RBD is shown in green and in blue ACE2. We show the location and sequence of three B cell epitopes that are in RBD or nearby. These B cell epitopes can be the targets of neutralizing antibodies.

SARS-CoV-2 entry in the host cells is facilitated by angiotensin-converting enzyme 2 (ACE2), which is engaged by the receptor binding domain (RBD) of the Spike protein (53, 54). Antibodies that interfere with such interaction have been shown to be neutralizing and block viral entry (29). Interestingly, two of the potential cross-reactive B cell epitopes (RLFRKSNL and SFELLHAPAT) from wP antigens map in solvent exposed regions of the spike RBD domain (**Figure 1**), indicating that they can be neutralizing. There is also a cross-reactive B cell epitope (FEYVSQPF) from a D antigen, which is in close proximity (15–20 Å) to the RBD domain. Antibodies targeting this epitope will, by steric hindrance, block viral engagement with ACE2. Other accessible B cell epitopes from D, T, and wP antigens could also lead to the same effect (**Table 6** and **Figure 1**). Antibody blocking of ACE2 interaction with Spike by steric hindrance is enhanced by the fact that SARS-CoV-2 Spike protein is a trimer.

We realize that we did not analyze if the potential cross-reactive B cell epitopes in SARS-CoV-2 are also accessible in the source proteins. Although such analysis can be of interest, we found it unnecessary since our approach was aimed to identify continuous/linear B cell epitopes and not-conformational B cell epitopes. Actually, all identified cross-reactive B cell epitopes in SARS-CoV-2 Spike protein with solvent accessibility above 33% map in loop regions. Moreover, it is worth noting that extracellular proteases can degrade antigens as well as immune cells such as neutrophils, which phagocyte antigens and release them semi-degraded. Thus, during the course of immune response there are all sort of antigen fragments (intracellular, extracellular, accessible, buried *etc*) and antibodies against them are generated, as shown by B cell epitope mapping analysis (55–57). Of all the antibodies generated against a given pathogen, only those recognizing accessible B cell epitopes in accessible antigens may be protective against that particular pathogen. However, our results support that all the remaining antibodies still can also be useful for providing cross-reactive immunity.

### DTP Vaccination and COVID-19 Epidemiology

Our data indicates that DTP vaccines can elicit ample T cell and B cell cross-reactive immunity against SARS-CoV-2. Likewise, MenB vaccines can also induce some substantial cross-reactive T cell immunity against SARS-CoV-2. However, MenB vaccines are unlikely responsible for the observed resilient of children to SARS-CoV-2 worldwide; MenB vaccines have been introduced very recently, in the last decade, and not everywhere (58, 59). In contrast, combination DTP vaccines are included in every single vaccination program since the 1940–50 (50). During infancy, children receive 3–4 doses (2, 3, 4, and 11 months of age) of either DTaP or DTwP, often in combination with inactivated poliovirus, conjugated Hib and recombinant antigens from HBV. Children also receive a pediatric DTaP booster vaccination at 4–6 years of age and a Tdap booster vaccination with lower antigenic load at 9–14 years of age. Moreover, pregnant women are also recommended to have a Tdap shot (https://www.who.int/topics/immunization/en/). Repeated vaccinations with DTP will therefore support that children and the youngest could be protected against SARS-CoV-2 through cross-reactive immunity elicited by these vaccines. Cross-reactive immunity elicited by DTP vaccines will eventually wane over time explaining why COVID-19 cases, and severity increases with age. We can also speculate that Tdap vaccination during pregnancy may also explain the lower incidence of COVID19 deaths in women. Moreover, Tdap vaccinated women could also pass passive cross-reactive immunity against SARS-CoV-2 to breast-feeding newborns.

**Figure 2:**
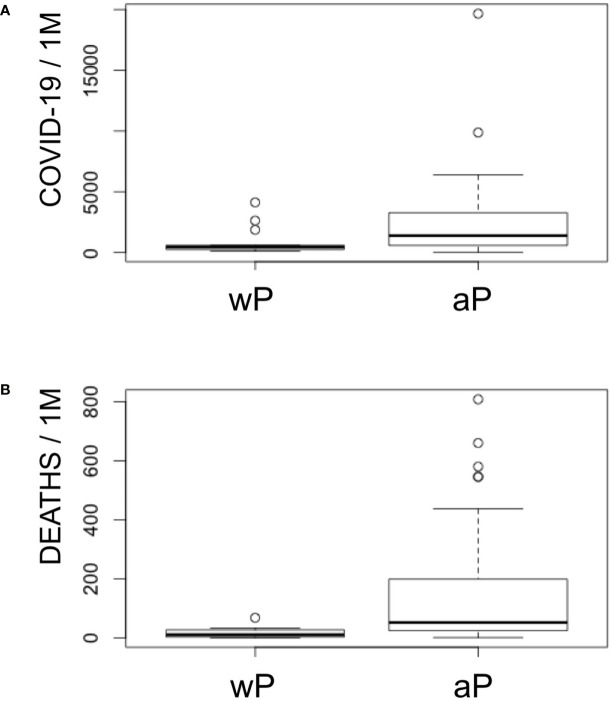
COVID-19 cases and deaths in Europe with regard to the P component in DTP vaccines. Panel shows a boxplot representation of COVID-19 cases **(A)** and deaths **(B)** per million in Europe including Asian countries from former URSS with regard to the inclusion of aP or wP in combination DTP vaccines. Non-parametric Kruskal–Wallis test indicates that both deaths and cases are significant lower in countries with DTwP vaccines (p < 0.005).

The current vaccination trend has favored the use DTaP instead of DTwP vaccines (50). In Europe most countries use combination DTaP vaccines, but some still use combination DTwP vaccines. Taken in consideration that the P component in DTaP vaccines has little contribution to the cross-reactive immunity SARS-CoV-2, we can also conclude that cross-reactive immunity from the D and T components is likely what protects children against COVID-19 worldwide. This protection may be enhanced by conjugated vaccines using D and T toxoids, such as Hib and PCV (45, 46, 48, 60). However, our data also indicate that cross-reactive immunity to SARS-CoV-2 from the P component in combination DTwP vaccines rivals that of D and T antigens together, suggesting that DTwP vaccines may confer more protection against SARS-CoV-2. Interestingly, COVID-19 deaths and cases per million in Europe and Asian countries of the former URSS (**Table S1**) are significantly lower (p < 0.005) in those countries implementing DTwP vaccines (**Figure 2**). Median deaths and cases per million in countries with wP vaccines are 13 and 451, while these same figures in countries with aP vaccines are 52 and 1,387.

Epidemiological data show that adolescents primed with DTaP during infancy are also less protected against clinical pertussis than DTwP-primed children (61). Priming with DTwP or DTaP combination vaccines during infancy actually determine later immune memory responses at preadolescent age to all DTaP antigens, with wP-priming resulting in a beneficial Th1-dominated response (62). Most likely this Th1-dominated response is also beneficial against SARS-CoV-2.

## Conclusion

Our findings indicate that cross-reactive immunity elicited by DT antigens in combination DTP vaccines is likely keeping children and the youngest safe from COVID-19 worldwide. Cross-reactive immunity elicited by these vaccines surely wanes over time explaining why COVID-19 cases and severity increases with age. The global health crisis caused by COVID-19 has prompted many efforts to develop a specific vaccine against SARS-CoV-2, which if successful will take a long time to achieve. However, we need a vaccine for SARS-CoV-2 today, and our results support that DTP vaccinations, and in particular DTwP vaccinations, could protect the general population against COVID-19 through cross-reactive immunity. Given the heterogenicity of DTP vaccines, our results also suggest that COVID-19 death rates and severity in different countries may be linked to differences in the composition of DTP vaccines, vaccination regimes, and initiation of DTP vaccination programs.

## Data Availability Statement

All datasets presented in this study are included in the article/**Supplementary Material**.

## Author Contributions

The author confirms being the sole contributor of this work and has approved it for publication.

## Conflict of Interest

The author declares that the research was conducted in the absence of any commercial or financial relationships that could be construed as a potential conflict of interest.

## Supplementary Material

Supplementary Data sheet 1 Excel file including all SARS-CoV-2 peptide matches and predicted cross-reactive epitopes in 25 human pathogens, including 18 viruses and seven bacteria.

Supplementary Data sheet 2 Excel file including all SARS-CoV-2 peptide matches and predicted cross-reactive epitopes from DTP and MenB vaccines.

Supplementary Table 1 COVID-19 cases and fatalities per million in different countries with regard to the use of aP/wP vaccines.
