# Supplementary material for: Potential Cross-Reactive Immunity to SARS-CoV-2 From Common Human Pathogens and Vaccines
Source: Front Immunol. 2020 Oct 16;11:586984. doi: 10.3389/fimmu.2020.586984 (PMC7596387; doi:10.3389/fimmu.2020.586984)
Supplement: Supplementary Table 1 — COVID-19 cases and fatalities per million in different countries with regard to the use of aP/wP vaccines. [file Table_1.pdf]

Table S1. COVID-19 cases and fatalities per million in different countries with regard to the use of aP/wP vaccines

Countries	wP		aP	
	Cases/ 10^6	Death/ 10^6	Cases 10^6	Death/ 10^6
Albania	365	11		
Andorra			9.877	660
Armenia	2.624	33		
Austria			1.843	72
Azerbaijan	451	5		
Belarus	4.122	23		
Belgium			4.971	808
Bosnia and Herzegovina			742	46
Bulgaria			354	19
Croatia			546	25
Cyprus			778	14
Czech Republic			845	30
Denmark			1.983	98
Estonia			1.387	50
Finland			1.208	56
France			2.8	437
Georgia	184	3		
Germany			2.167	101
Greece			277	17
Hungary			393	52
Iceland			5.293	29
Ireland			5.015	327
Israel			1.823	31
Italy			3.813	545
Kazakhstan	496	2		
Kyrgyzstan	233	2		
Latvia			560	12
Lithuania			604	24
Luxembourg			6.393	176
Malta			1.386	16
Monaco			2.499	102
Montenegro			516	14
Netherlands (the)			2.672	343
North Macedonia			979	57
Norway			1.549	43
Poland	589	27		
Portugal			3.068	133
Republic of Moldova (the)	1.868	68		
Romania			966	64
Russian Federation (the)	2.54	27		
San Marino			19.662	1.238
Serbia			1.29	27
Slovakia			278	5
Slovenia			708	52
Spain			6.06	580
Sweden			3.476	418
Switzerland			3.559	222
Tajikistan	360	5		
Turkey			1.884	52
Ukraine	501	15	501	15
United Kingdom of Great Britain and Northern Ireland (the)			3.909	546
Uzbekistan	100	0.4		

Data collected from https://www.worldometers.info/coronavirus/ on May 27th 2020.
wP/aP vaccination obtained from https://apps.who.int/immunization_monitoring/
